# Supplementary material for: Curcumin-Loaded Solid Lipid Nanoparticles Bypass P-Glycoprotein Mediated Doxorubicin Resistance in Triple Negative Breast Cancer Cells
Source: Pharmaceutics. 2020 Jan 24;12(2):96. doi: 10.3390/pharmaceutics12020096 (PMC7076516; doi:10.3390/pharmaceutics12020096)
Supplement: Supplementary file 1 [file pharmaceutics-12-00096-s001.pdf]

pharmaceutics

Supplementary Materials: Curcumin-Loaded Solid Lipid Nanoparticles Bypasses P-glycoprotein Mediated Doxorubicin Resistance in Triple Negative Breast Cancer Cells

Gamal-Eldein Fathy Abd-Ellatef, Elena Gazzano, Daniela Chirio, Ahmed Ragab Hamed, Dimas Carolina Belisario, Carlo Zuddas, Elena Peira, Barbara Rolando, Joanna Kopecka, Mohamed Assem Said Marie, Simona Sapino, Sohair Ramadan Fahmy, Marina Gallarate, Abdel-Hamid Zaki Abdel-Hamid, Chiara Riganti

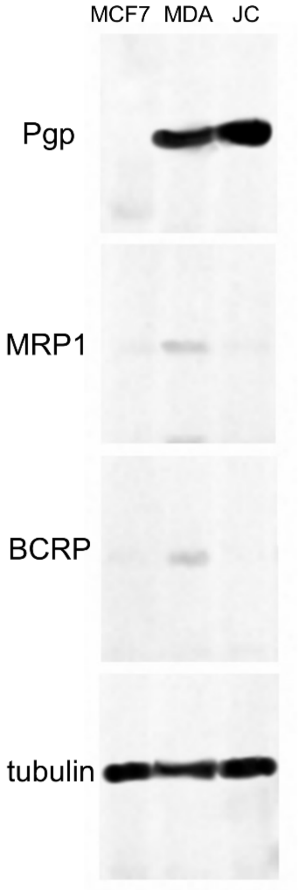

Figure S1. Expression of Pgp, MRP1 and BCRP in the cell lines analyzed. Human breast cancer MCF7 and MDA-MB-231 cells, and murine JC cells were lysed and probed with the indicated antibodies. Tubulin was used to check the equal loading of proteins. The figure is representative of one out of three experiments.

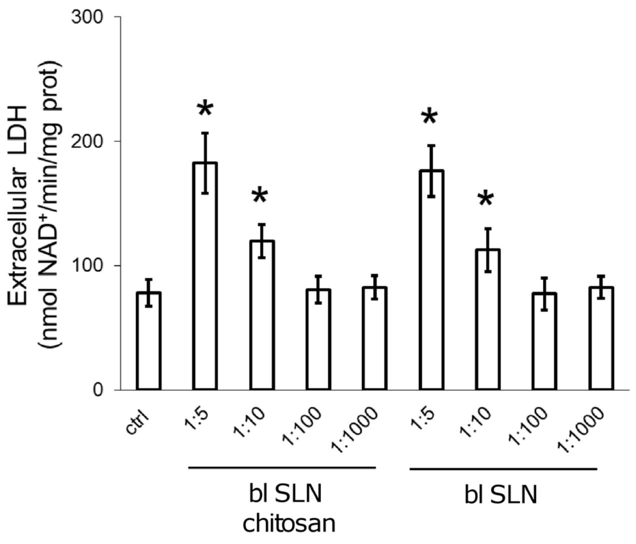

Figure S2. Dose-dependent cytotoxicity of blank SLNs in MDA-MB-231 cells. MDA-MB-231 cells were incubated 24 h in fresh medium (ctrl) or in medium containing blank SLN (bl SLN), with or without chitosan, diluted 1:5, 1:10, 1:100, 1:1000 in the culture medium. The release of LDH in the extracellular medium was measured spectrophotometrically in duplicates. Data are presented as means ± SD (n = 3). * $p < 0.01$: vs untreated cells (ctrl).

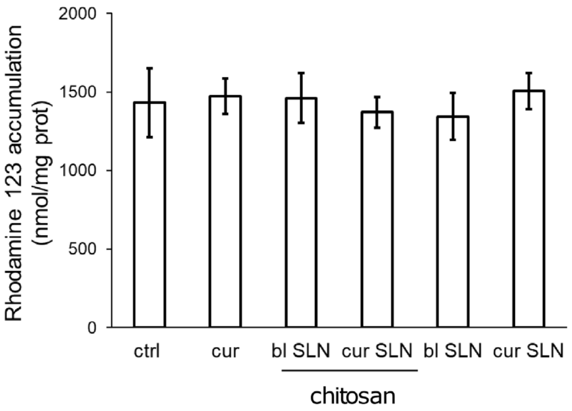

Figure S3. Effects of CURC-loaded SLNs co-incubated with rhodamine 123 on the dye retention. MDA-MB-231 cells were co-incubated for 20 min with fresh medium (ctrl), blank SLN (bl SLN), CURC-loaded SLN (cur SLN, containing 5 μM cur), with or without chitosan, together with rhodamine 123. The intracellular accumulation of rhodamine 123 was measured fluorimetrically in duplicates. Measurements were performed in triplicate and data are presented as means ± SD (n = 3).

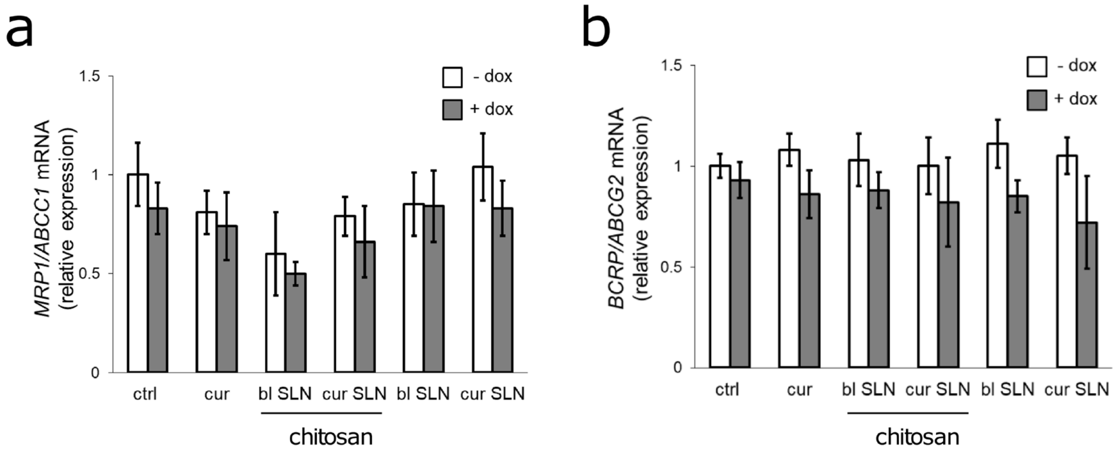

Figure S4. Effects of CURC-loaded SLNs on MRP1 and BCRP expression. MDA-MB-231 cells were incubated 24 h with fresh medium (ctrl), 5 μM CURC (cur), blank SLN (bl SLN), CURC-loaded SLN (cur SLN, containing 5 μM cur), with or without chitosan. When indicated, 5 μM doxorubicin (dox) was added. The expression of the MRP1 (panel A) and BCRP (panel B) mRNAs was measured by qRT-PCR in triplicates. Data are presented as means ± SD ($n = 3$).

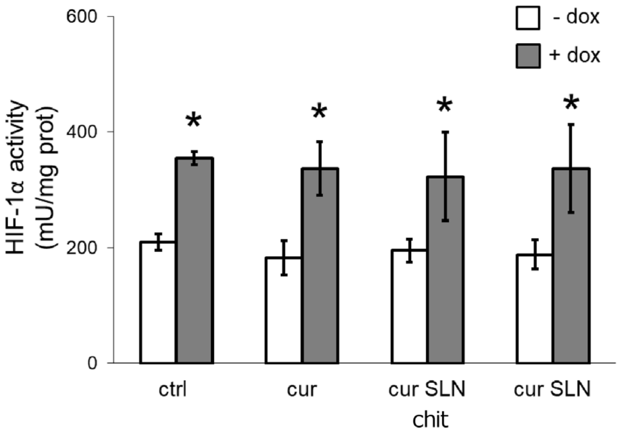

Figure S5. Effect of CURC-loaded SLNs on HIF-1α activation. MDA-MB-231 cells were incubated with fresh medium (ctrl), 5 μM CURC (cur), CURC-loaded SLN (cur SLN, containing 5 μM cur), with or without chitosan. When indicated, 5 μM doxorubicin (dox) was added. HIF-1α activation was measured by ELISA in triplicates. Data are presented as means ± SD ($n = 3$). * $p < 0.001$: vs ctrl.

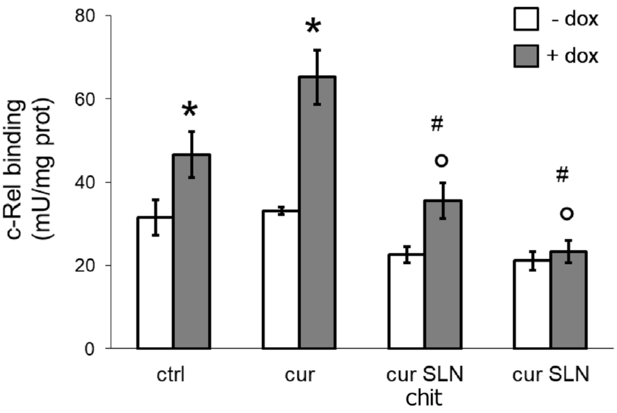

Figure S6. Effect of CURC-loaded SLNs on c-Rel activation. MDA-MB-231 cells were incubated with fresh medium (ctrl), 5 μM CURC (cur), CURC-loaded SLN (cur SLN, containing 5 μM cur), with or without chitosan. When indicated, 5 μM doxorubicin (dox) was added. The binding activity of c-Rel was measured by ELISA, in duplicates. Data are presented as means ± SD ($n = 3$). * $p < 0.001$: vs ctrl; ° $p < 0.02$: vs dox; # $p < 0.01$.
